# Content Analysis of Media Coverage of Childhood Obesity Topics in UAE Newspapers and Popular Social Media Platforms, 2014-2017

**DOI:** 10.15171/ijhpm.2018.100

**Published:** 2018-11-21

**Authors:** Niyi Awofeso, Sara Al Imam, Arwa Ahmed

**Affiliations:** School of Health and Environmental Studies, Hamdan Bin Mohammed Smart University, Dubai, UAE.

**Keywords:** Media Content Analysis, United Arab Emirates, Periodicals, Pediatric Obesity, Social Media

## Abstract

The 2017 prevalence of obesity among children (age 5–17 years) in the United Arab Emirates (UAE) is 13.68%. Childhood obesity is one of the 10 top health priorities in the UAE. This study examines the quality, frequency, sources, scope and framing of childhood obesity in popular social media and three leading UAE newspapers from 2014 to 2017. During the review period, 152 newspaper articles from three leading national newspapers – Gulf News, The National and Al Ittihad – met the eligibility criteria for this study. There were 57 Facebook posts, 50 Twitter posts, 14 posted YouTube videos, and 13 Media releases on related to childhood obesity between 2014 and 2017. Childhood obesity was consistently problematized, primarily in health terms, but was not strongly linked to socio-economic and geographical factors. Childhood obesity was framed as being predominantly influenced by individual and parental behaviours more frequently (n = 76) compared with structural or environmental factors such as the roles of the food and beverage industry (n = 22). Unlike findings from studies with adult obesity, articles advocating individual behavior changes to address childhood obesity were relatively few (n = 29). Social media may be an effective way to help children overcome obesity, in part through online interaction with health care providers and health conscious obese peers. Areas for improvement in social media use to reduce childhood obesity prevalence in UAE include enhancing public engagement with social media posts on childhood obesity, as reflected in the numbers of Likes and Retweets or Shares.

## Introduction

According to World Health Organization (WHO) guidelines, an obese child is one whose body mass index (BMI) is greater than 95% of BMIs of children of the same age and gender in the reference population. WHO defines children from age five to less than 19 years with BMI-for-age more than 2 SD above the WHO growth reference median.^1^ The October 2017 report on ending childhood obesity stated that, globally, 124 million children and adolescents are obese – a tenfold increase in the last 4 decades.^2^ In the United Arab Emirates (UAE), the 2017 prevalence of obesity among children (age 5–17 years) was 13.68%, according to the UAE Vision 2021 performance indicators for world class health services. This prevalence is higher than that of any nation in the European Union (EU) or the EU 2013 average prevalence for childhood obesity of 6.6%.^3^ Obese children are more likely to develop a variety of health problems as adults. These include cardiovascular disease, insulin resistance (often an early sign of impending diabetes), musculoskeletal disorders (especially osteoarthritis), and some cancers (endometrial, breast, and colon). In the United States, researchers estimate the incremental lifetime direct medical costs of an obese child relative to a normal weight child to be between $12 660 and $19 000.^4-6^

The mass media’s role in childhood obesity is ambiguous. Excessive media engagement, exemplified by TV installation in children’s bedroom is commonly considered a feature of obesogenic environments. Children inhabiting obesogenic environments have been shown to have reduced opportunities for physical activity as well as inadvertent exposure to advertisement of unhealthy foods.^7^ On the other hand, the mass media, and social media in particular, may contribute positively to reducing childhood obesity though effective dissemination of credible information among key stakeholders, and sustaining informational social influence when one member of an obese adolescent cohort begins to demonstrate effective weight conscious behavior.^8^ Parents have a major influence on children’s weight profiles, in part through acculturation, as well as through accessing and adapting credible health information related to childhood obesity in newspapers and social media to family settings. Although parents are one of the main influences when it comes to shaping their children’s decisions in relation to healthy eating. Some parents with low health literacy, may have inadequate knowledge about food and diet, and may not know how to translate this knowledge into decisions about healthy eating. Tactfully considering children’s views in food choices may enhance acceptance of healthy food choices. While children eat most foods at home, health authorities devote more attention to school meals than to supporting mothers to provide healthy meals and minimize obesogenic environments.^9,10^

The UAE community’s level of trust in government-supported newspapers is very high, with 87% of 2500 respondents in a stratified sampling survey stating that they trust the veracity of health information articles in local newspapers. About 81% of survey respondents trust online government-supported or approved media outlets and news portals.^11^ In 2017, 79% of UAE’s population – young and old – regularly accessed the Internet. Facebook is the most commonly used social media platform, with 81% of the population registered as Facebook users.^12^ Government health authorities use Twitter more commonly to share information than any other social media platform. Compared with the volume of studies that have identified a detrimental role of newspapers and social media in childhood obesity incidence and stigmatization, relatively few studies have explored social media’s actual and potential roles in facilitating obesity reductions.^13^ For instance, fairly common reporting on obesity as a public health crisis brought on by bad personal choices can worsen anti-fat prejudice in accessing needed health services and stigmatization.^14^ Relatively uncommon, however, are mass media reports about anti-fat stigma or the role of newspapers and social media in reducing childhood obesity.^15,16^ Newspaper advertisements and articles related to childhood obesity are more likely to influence UAE parents’ attitudes and behaviours than children’s , since relatively few UAE children read newspapers. The limited exposure of children to newspapers is due in part to more appealing alternatives such as social media, and in part to the low priority accorded children (eg, by interviewing them) in newspaper articles.^17^ Nevertheless, parental control, attitudes, and behavior in relation to childhood obesity are key variables in developing prevention programs.^18^

Social media is a novel context for social network dynamics and social learning. However, child feeding and physical activity topics and influence of social media on the manner in which mothers’ implement children’s feeding and physical activity practices are sparse.^19^ While very few children access social media sites to seek health information, young people appear to be accommodating of fast foods advertising on popular social media platforms such as Facebook.^20^ Many leading fast food brands engage in “below the belt” advertising which implicitly target children. For example, surreptitious advertising of many video logs (vlogs) found on YouTube and Instagram sites are produced by media influencers with large following of young people who have financial interests with multinational food and drinks corporations. Such vlogs can have a positive influence on the brand attitude and willingness to purchase.^21^ Concerning child obesity, social media operates as a double edged sword in the sense that it may both influence the development of obesity and serve as a vehicle for its prevention and management.^22,23^

Determinants of childhood obesity vary by context. In the UAE, childhood obesity is commonly associated with obesogenic environments – described as the sum of influences that the surroundings, opportunities, or conditions of life have on promoting obesity in individuals or populations.^24^ Behavioural and socialization factors which influence childhood obesity in UAE and neighboring nations include increased consumption of calorie-dense food, infrequent breakfasts, and a sedentary lifestyle, including not travelling actively to school.^25,26^ Unlike the situation in many Western nations, educated, working mothers are significantly more likely to have obese children in Middle Eastern nations. Working mothers in wealthy Arab nations tend to have less time available for preparing food at home, which leads to consumption of fast foods and energy dense meals prepared at restaurants.^27^

Policy decisions on public health challenges such as childhood obesity areas influenced by opinion leaders, whose perspectives are commonly influenced by mass and social media representations and/or prioritization of such issues. These media play important roles in setting the public agenda, framing issues of public interest, attribution of causes and agents, victims and possible solutions.^28^ Mainstream and social media publicity of health-related topics have an influential effect on the public’s knowledge and awareness of health issues and may therefore have the potential to promote positive behaviour change.^29^ Kininmonth et al’s 2017 study^30^ of the quality of 141 nutrition articles in 5 of the 6 highest circulating British newspapers revealed that 44 (31%) of reviewed articles were of poor quality. Another UK-based study of health-related articles in eight popular British newspapers identified 160 articles over a 2-month period.^31^ These studies indicate high frequency of health related, and in particular obesity related, articles in UK-based newspapers. Many government agencies in developed nations provide news reports on childhood obesity with varying levels of frequency. In the United Kingdom, the National Health Service (NHS) choices site publishes at least one child obesity related article every month in its “Behind the Headlines” section. This page is professionally edited and crafted to provide scientific information in a format that is reader-friendly, such as the review of a study on the effectiveness of school-based programs for addressing childhood obesity.^32^

Despite a rising popularity in the use of online media to access health information in most nations, printed newspapers remain an efficient and traditional way of providing the public and especially health policy makers with essential information to enable them to make informed decisions.^33^ The stakeholders who contribute to newspaper reporting on obesity strongly determine the quality and frame of such articles. In Kininmonth et al’s study^30^ of quality of nutrition-related articles in leading UK newspapers, particularly low-quality scores were obtained for anonymously published articles with no named journalist, articles that focused on childhood obesity, and articles that reported on high fat and processed foods. Similarly, Robinson et al^31^ and Kininmonth et al^34^ found that anonymous articles on health related topics had the poorest quality. In addition, the authors determined major differences in the quality of reporting within and between leading daily UK newspapers, with highest quality articles were in The Times and The Independent, with the lowest quality articles in The Sun. Importantly, UK newspapers rely heavily on press releases from major UK scientific journals. Such over-reliance might narrow reporting scope and limit objective reporting, especially since quality of journal press releases is not uniformly high.^35^ Dietary advice, which is pertinent to childhood obesity prevention is commonly inaccurately reported in newspapers even in industrially advanced nations. In many nations, newspaper reporting of childhood obesity related topics has often been sensationalist, with the headlines not accurately reflecting the scientific research, and based on reporting preliminary research as a ‘breakthrough.’

Social media platforms are increasingly resorted to by youth and young parents to access health information. For example, a 2016 study of 7840 participants in 7 nations, 52% used websites to access health information, 38% used mobile/tablets for health matters, 27% used social media, and 16% used online support communities.^36^ Not surprisingly, government agencies are increasingly motivated to establish social media platforms in order to connect better with critical demographics in society especially on issues such as childhood obesity. Social media can both help facilitate information sharing and be problematic in spreading rumors (“fake news”) during seasonally expected health events and health crises. Childhood obesity is a chronic health crisis in many nations, with potential financial spinoff for charlatans who propagate unproven remedies for financial gain. It is therefore crucial that public health agencies and organizations are equipped with effective social media communication strategies, tools and workforce to both provide accurate information, engage productively with parents and other stakeholders on childhood obesity issues, and counter misinformation related to this topic on social media. As a minimum, health departments and ministries should have a social media policy (and strategy), an up-to-date internet webpage and at least one profile in popular social media networks such as Facebook and Twitter. In efforts to address childhood obesity, social media use should be incorporated into most health communication strategies.^37^

This study examines the quality, sources and coverage of three leading UAE newspapers’ coverage of childhood obesity between 2014 and 2017 and examines how obesity is framed in terms of defining the problem, attributing causes and presenting solutions. In addition, the social media posts of three major health authorities in UAE are reviewed for content, views, likes and shares or retweets.

The research questions that this study seeks to answer are:

1. How frequently are childhood obesity articles and posts focused on UAE published in the three leading UAE newspapers and by the three main health authorities in UAE?
2. Which stakeholders contribute newspaper articles related to childhood obesity, and how are the contents of such articles framed?
3. What is the quality and coverage of childhood obesity articles and posts by the three main health authorities in the UAE in Twitter, YouTube, and Facebook?

## Methods

We utilized content analysis methods for this study. We used quantitative content analysis to measure the frequency of content within the articles across the whole sample, coupled with qualitative content analysis to facilitate categorization of the articles by source and content.^38^ A document may be described as any symbolic representation that can be recorded or retrieved for analysis, and includes social media sites and newspapers. For the purpose of this study, three well-regarded nationally circulated newspapers and three popular social media sites in UAE were selected as sources for the documents related to childhood obesity media activities in UAE. The National is an Abu Dhabi based newspaper with national circulation which has been in continuous publication since April 16, 2008. It covers local and international news, business, sports, arts and life, travel and health matters. It is pre-dated by a sister Arabic language newspaper publication titled Al Ittihad (ie, “Union”), which has been published continuously since October 20, 1969. The Gulf News is a Dubai-based daily national newspaper, first launched in tabloid format in 1978. It has a circulation of about 100 000 daily, and is UAE’s most accessible newspaper.

The review period for this study is between January 1, 2014 and December 31, 2017. For newspaper articles, classification was made based on the sources of the news reports (eg, expert opinions, and civil society official policy documents) as well as the contents (eg, Individual behavioral changes proposed, and opinions linked to the roles of the food and beverage industry). A triangulated search approach was adopted, whereby the keywords “Health,” “Childhood obesity,” “Children and Health,” “Overweight,” “Fat,” “Social Media and Obesity,” “DHA and Obesity,” “DHAD and Obesity,” and “MoHAP and Obesity” were searched for in the search engines of the three newspapers between June 2017 and January 2018, and all hits were read and analysed for eligibility for inclusion as well as for classification if eligible. Eligible articles were reviewed for possible classification as Alarmist, Reassuring or Neutral. We operationalized alarmist articles are those which exaggerate the significance or consequences of a reported childhood obesity issue or study (eg, “Obese children likely to die up to 20 years earlier than healthy peers” https://www.independent.co.uk/life-style/health-and-families/obesity-children-death-premature-life-expectancy-comparison-report-warning-a8173921.html). We defined reassuring articles as those that provide practical information, research reports or case studies on how childhood obesity is being successfully addressed in UAE or internationally. Neutral reports were described as those which simply reported interviews of opinion leaders, childhood obesity programs or research reports without offering opinions as to the merits or evaluation of reported news. The thematic content of articles was examined using manifest content analysis. A total of 152 eligible articles published between January 1, 2014 and December 31, 2017 were retrieved from the respective newspapers’ electronic databases using keyword searches.

A search for similar items was conducted on accounts held by Dubai Health Authority (DHA), Ministry of Health and Prevention (MoHAP), and Department of Health Abu Dhabi (DHAD) on three popular social media platforms – Facebook, YouTube and Twitter. The three health agencies, UAE MoHAP (oversees all Emirates as well as provides local services to the northern emirates of Sharjah, Ajman, Ras Al Khaimah, and Oum Al Quain ), DHAD (formerly Health Authority Abu Dhabi – HAAD – covers Abu Dhabi Emirate) and DHA (covers Dubai Emirate) are collectively responsible for health care policy making and regulation as well as delivery of publicly funded health care services within UAE.

Eligible social media posts were categorized by content, number of views, number of likes, and number of shares or retweets. The contents of all eligible articles were studied, and Bacci’s method of problem representation^39^ was utilized in order to classify the articles. Carol Bacci’s approach to media analysis is based on the premise that the framing of media reports on childhood obesity is based on what authors would like policy makers to do about it.

We used the same keywords related to childhood obesity that were used in the newspapers search engines earlier in the three social media platforms: Facebook, Twitter, and YouTube; in which, we found only few of the results, since the social media result of searches prioritize the recent posts more than old posts from 4 years ago, that are less relevant. Therefore, we conducted a second phase of search through the channel or page of the health authority itself in these platforms. In this phase, results were narrower, searching manually for childhood obesity related topics, posts, or videos in DHA/MoHP/DHAD pages, providing more results that were missed in the first phase. By separating the core primary messages from posts and comparing their times of publishing, we have implicitly avoided duplicates. In the data collection step, we have listed all the results we retrieved from the platforms, and ordered them in terms of authorities responsible, and date of posting. Evaluation of eligible articles for inclusion in the study entails establishing the relationships of tweets, posts and videos to the topic under review – eg, by link to specified keywords and evaluation of video contents/tweets/posts. All posts/tweets/videos are subsequently analysed for viewer engagement as measured by re-tweets, shares and/or likes whenever applicable.

We based our classifications of media articles in newspapers largely on how we surmised ‘problems’ related to childhood obesity were constituted by the newspaper reports. We operationalized ‘quality’ in our manuscript as content quality, based on the extent to which an article published on a social media platform published by UAE government departments is perceived in terms of at least several of the following attributes: user feedback frames; appropriateness of volume of data; objectivity – extent to which information is unbiased, unprejudiced and impartial; relevance – extent to which information is applicable and helpful for the task at hand; reliability – extent to which information is correct and reliable; comprehensibility – extent to which data are clear without ambiguity and easily comprehended, and; value-added – extent to which information is beneficial, provides advantages from its use. We selected social media articles that meet at least three of these attributes in the study.

**Figure 1:**
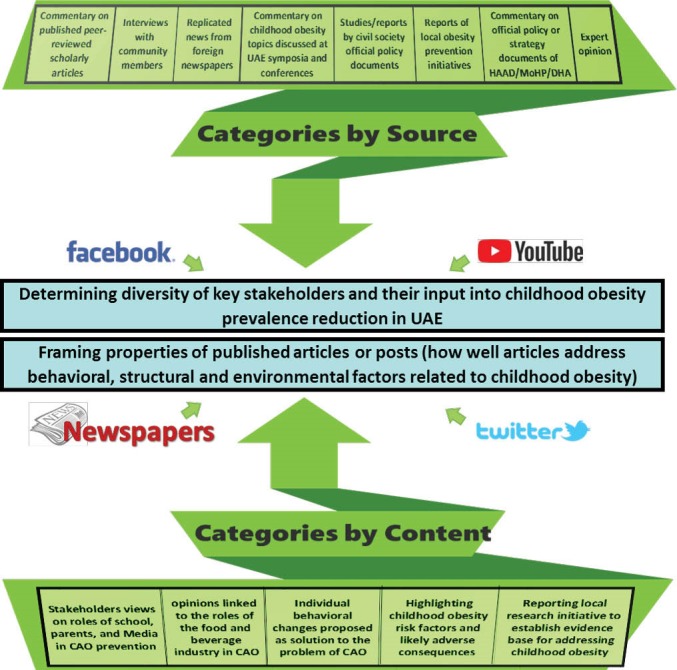
Conceptual Framework for Mass Media and Childhood Obesity.

The conceptual framework for this study is based on the study of media reports related to sources of information and content of the information, in order to determine key stakeholders and their influence on government efforts to reduce childhood obesity prevalence in UAE. In addition, we sought to determine framing properties of published articles or posts, how well articles address behavioural, structural and environmental factors related to childhood obesity, and the potential readership of such articles within UAE (Figure 1).

## Results

*RQ1: How frequently are childhood obesity articles and posts focussed on UAE published in the three leading UAE newspapers and by the three main health authorities in UAE *?

The average yearly number of articles focussed on childhood obesity (38 per year) is comparable to newspaper reports on this topic in Australia^40^ and the United Kingdom.^41^ For the Australian example focussed on obesity among indigenous populations between 2007 and 2014, 38 articles were extracted during the review period. Given that indigenous Australians constitute a key population with rising obesity rates, it is surprising that, on average, about 5 articles per year were published about this topic during the review period. In contrast, for the British study on obesity reporting in the United Kingdom over a 15-rear period in 7 newspapers, 2414 articles were deemed eligible (160 articles per year) of which 40% (64 per year) were related to child obesity. We believe that compared with UK’s mature newspaper readership and culture, 38 childhood obesity related publications per year in 3 newspapers compares favourably with 64 publications per year in 7 newspapers.

The high proportion of reporting of UAE nutrition experts’ views on childhood obesity is noteworthy as it shows that journalists are keen to interview and report the perspectives of a wide array of specialists in this field. However, there is relatively sparse reporting of expert opinions on published articles and best practices on national and international initiatives to address childhood obesity, such as Finland’s remarkable progress in addressing childhood obesity and the recently released action plan to address childhood obesity in the United Kingdom.^42,43^ In the 33 commentary reports on scholarly articles related to childhood obesity found during the review period, details of the primary sources of evidence, such as links to pertinent journal articles, were not included in the report.

*RQ2: Which stakeholders contribute newspaper articles related to childhood obesity, and how are the contents of such articles framed *?

**Figure 2:**
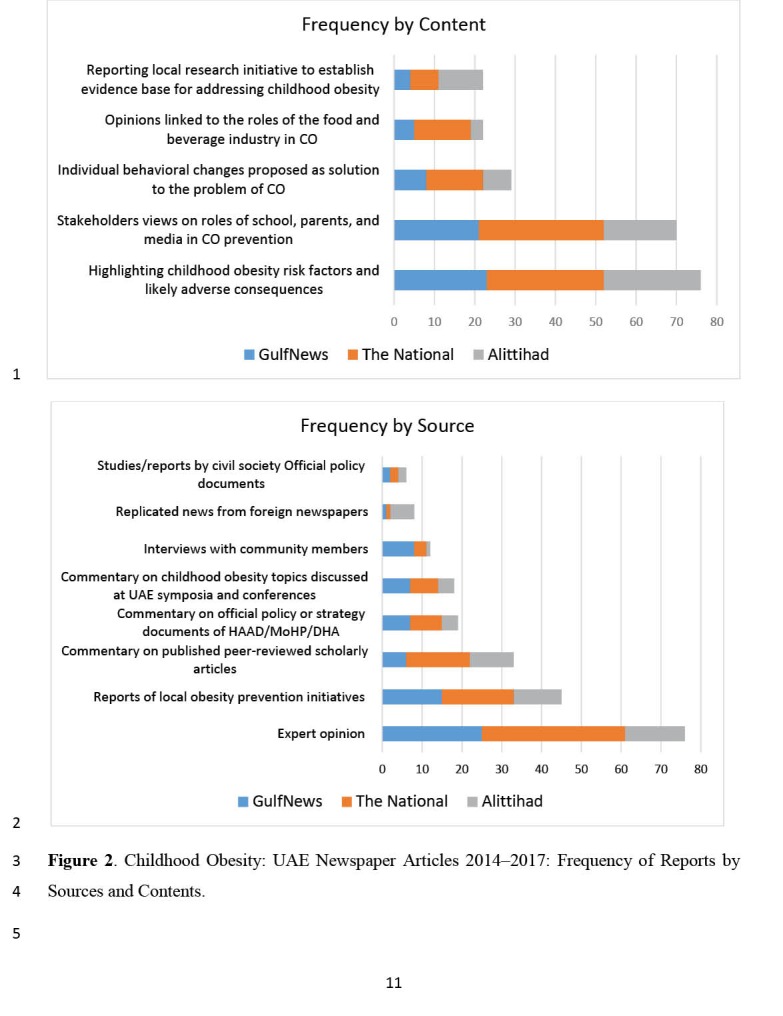
Childhood Obesity: UAE Newspaper Articles 2014–2017: Frequency of Reports by Sources and Contents.

Data on categories by sources of information and categories by content characteristics for the published articles are shown in Figure 2.

The general trend, (with the exception of Al Ittihad newspaper articles on the subject, which peaked in 2016) has been towards increased reporting of childhood obesity newspaper articles. The vast majority of the headlines and contents of the reports (86%) were coded as neutral in the sense that they largely factually reported on health related aspects and initiatives to address childhood obesity, while the remainder were classified as reassuring. Alarmist reports on childhood obesity are very rare in UAE newspapers, with obesity largely framed as an unanticipated consequence of improving socio-economic development in UAE, for which effective medical and policy measures exist.^44^

The framing of childhood obesity in UAE newspapers is appropriately geared towards agents of socialization – parents, schools, peers – and government policies, rather than on encouraging children to change dietary and exercise behaviours. However, only 6 of the 29 newspaper reports related to behavioural interventions to address childhood obesity focussed on important behaviour related influences such as individual and parental behaviours towards obesogenic environments, practices such as reducing food portion sizes^45^, counselling^46^ or modifying food rewards culture.^47,48^

Media characterizations of and reporting on obesity policy is generally inadequate. This is reflected in the generally superficial 19 commentary articles on UAE health authorities’ policies and programs related to childhood obesity over the review period. For example, although limited information newspaper reports indicate that multifaceted and intersectoral approach being adopted by the panel established in November 2017 to tackle childhood obesity in Abu Dhabi is similar to those successfully applied in Finland,^48^ no official Abu Dhabi obesity strategic plan is currently available for critique.

*RQ3: What is the quality and coverage of childhood obesity articles and posts by the three main health authorities in the UAE in Twitter, YouTube, and Facebook *?

**Figure 3:**
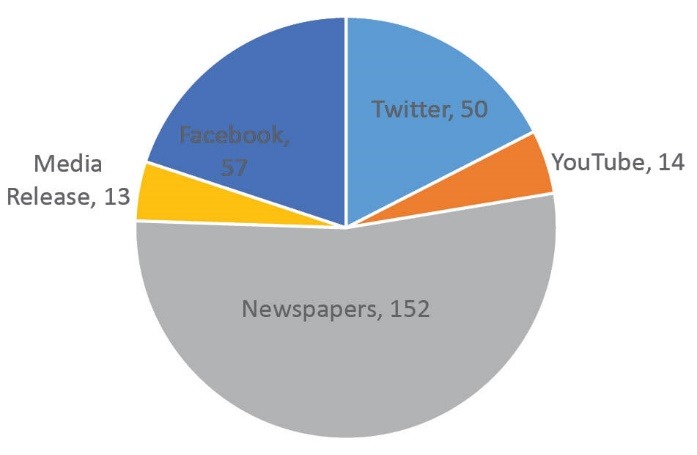
Distribution of Childhood Obesity Topics in Print and Social Media in UAE Between 2014 and 2017.

The coverage of childhood obesity articles by YouTube, Twitter, Facebook and online media releases numbered 134, compared with 152 newspaper articles on childhood obesity during the review period (Figure 3).

**Figure 4:**
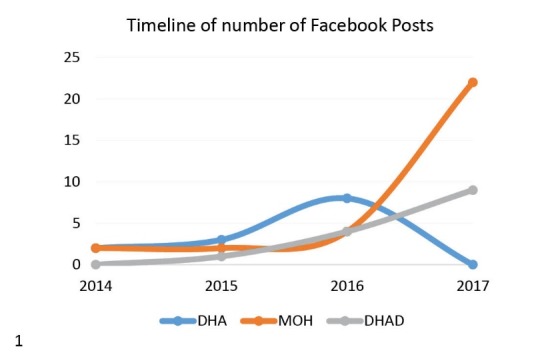
Trends in Facebook Posts by UAE Government Health Authorities From 2014 to 2017.

Review of Facebook platform use by the three main health authorities in UAE to disseminate childhood obesity messages – Facebook, Twitter, and YouTube – revealed relatively modest but generally improving engagement with social media on childhood obesity issues, with the Federal Ministry of Health playing a more active role in social media circles. For Facebook posts by UAE government agencies, these trends are indicated in Figure 4.

**Figure 5:**
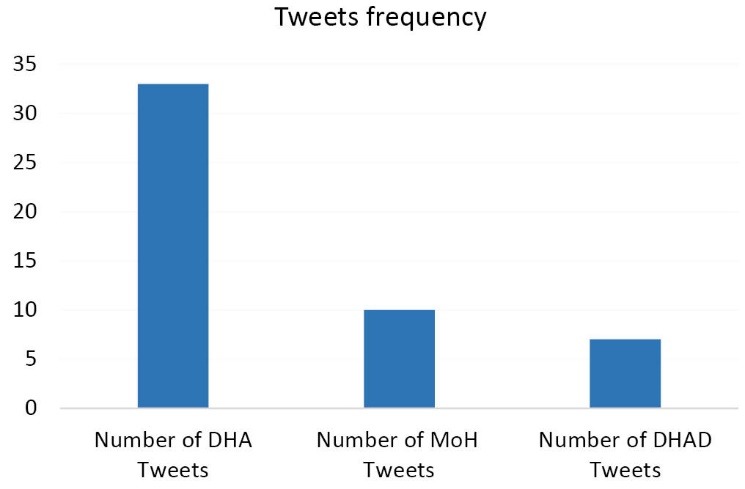
Tweets’ Frequency on Child Obesity Topics by UAE health Authorities.

The frequency of tweets on childhood obesity by UAE health authorities during the review period are shown in Figure 5.

With regards to YouTube platform, a combined total of 14 videos on childhood obesity were produced during the review period, with the MoHAP being the most active in this platform. It is noteworthy that YouTube was not used by any of UAE’s major health agencies in 2014. The number of views (n = 10 193), likes (n = 502), and shares (n = 9) of social media posts and media releases on childhood obesity by government agencies indicate low public engagement with social media posts by government health agencies on this topic.

Table shows details of YouTube posts, including views, likes and shares/retweets by the three UAE health authorities during the review period.

**Table:** YouTube Posts by UAE’s Leading Health Authorities

**Social Media Channel**	**Authority**	**Video Link**	**Title**	**Date**	**No. of Views**	**No. of Likes**	**Total Views**
YouTube	DHA	https://www.youtube.com/watch?v=H7dJdSHC0CU	Doctor Advice about childhood obesity risk factors	15-Mar-2017	46	0	297
YouTube	DHA	https://www.youtube.com/watch?v=jcAUIXSkl94	Doctor Advice about video games and relation to health and obesity	15-Mar-2017	55	0	
YouTube	DHA	https://www.youtube.com/watch?v=N5m8rWxb208	Doctor Advice about physical activity	23-Sep-2016	73	0	
YouTube	DHA	https://www.youtube.com/watch?v=6TxJr3cUvb0&t=2s	Medical Advice about childhood obesity	30-Apr-2016	123	2	
YouTube	DHAD	https://www.youtube.com/watch?v=VleLkdTxeCI	Children Exercise	8-Feb-2016	211	0	278
YouTube	DHAD	https://www.youtube.com/watch?v=6fs3wS5uHdw	Healthy Breakfast for children	8-Feb-2016	67	0	
YouTube	MoH	https://www.youtube.com/watch?v=4RRdcHGK9NI	Health Heroes app at Children City	21-Nov-2017	5	0	9255
YouTube	MoH	https://www.youtube.com/watch?v=LwNhso732Zk	Health Heroes app at Jalila Center	15-Nov-2017	18	0	
YouTube	MoH	https://www.youtube.com/watch?v=H_mQkxyYmUk	Childhood and Adolescents Obesity Prevention Workshop	24-Jul-2017	3821	1	
YouTube	MoH	https://www.youtube.com/watch?v=asERC9oOw_M	Health Heroes App Characters	28-Nov-2016	1850	14	
YouTube	MoH	https://www.youtube.com/watch?v=F5rJv-El4v4	Launching of Health Heroes App	28-Nov-2016	16	0	
YouTube	MoH	https://www.youtube.com/watch?v=1EnntlYioV4	MoH department of health education and promotion participating in Festival for children's health and Fitness	27-Feb-2016	2665	1	
YouTube	MoH	https://www.youtube.com/watch?v=UzCRRJTOAjo	Our Healthy Children Campaign	7-Nov-2015	479	5	
YouTube	MoH	https://www.youtube.com/watch?v=YncFVoYOK2Y	Awareness activities for children for Our Healthy Children Campaign	6-Nov-2015	401	1	
Total Number of YouTube Videos = 14							

Abbreviations: UAE, United Arab Emirates; DHA, Dubai Health Authority; MoH, Ministry of Health; DHAD, Department of Health Abu Dhabi.

## Discussion

Policy decisions are influenced by perceived public opinion, which is in turn influenced by mass media representations of issues. Newspapers are a well trusted mass media variety in UAE. Agenda setting according to Wu and Coleman^49^ is a “phenomenon of the mass media selecting certain issues and portraying them frequently and prominently, which leads people to perceive those issues as more important than others. Media representations of childhood obesity contribute to policy makers’ and public understanding of the obesity issue, including its drivers and solutions, and such perspectives may influence public acceptance of legislative interventions.^50^ The overwhelmingly neutral reporting on obesity in UAE newspapers (no articles with overtly alarmist slant or those focussed on child obesity and stigma or discrimination were found during the review period) suggest a belief among UAE journalists of children being “innocent victims” of obesity.^50^ A recent UAE public opinion survey of public interest in health stories in general in 2 English language newspapers including Gulf News indicated that 22% of respondents would like to read about health stories, ranking it sixth in the priority list of stories of interest to the newspapers readers. Correspondingly, with 1.3 health related articles per issue, health-related topics rank 6^th^ most frequently reported news category in the Gulf News.^51^ This implies a correlation between public demand for health news and the agenda setting processes of Gulf News management. However, given health topics’ relatively low ranking compared with local crime, social issues and education topics, there is room for improvement in raising the profile of health news in UAE news media. The increasing coverage of childhood obesity issues in UAE newspapers is consistent with increasing information on the health and economic consequences of this public health challenge.

The newspapers can play a more proactive role on childhood obesity by providing added value to reports through greater detail (eg, of health and economic consequences), guiding readers to primary sources of scholarly articles reviewed, in-depth analysis of government policies and programs on child obesity, and sharing lessons on international best practices on obesity prevention with UAE policy makers, parents and other agents of socialization of children.^52^

Concerning social media use in child obesity by UAE government health authorities, the sophistication, coverage and scope lags behind those of most Western nations. For example, although Twitter is widely used for diverse purposes by the Dubai Health Authority, DHAD, and the MoHAP, only 50 Twitter posts related to childhood obesity were available during the review period. Social media platforms are more practical and effective for directly engaging with children and parents compared with newspapers. Given the high social media penetration in UAE, opportunities abound for utilizing this platform to address childhood obesity, as is currently the case in the United Kingdom, United States, and Scandinavian nations.

Evaluation studies on child obesity campaigns using social media indicate that they were more effective in raising awareness than in consistently changing attitudes and behaviours that affect childhood obesity.^53^ The MoHAP has officially launched the Health Heroes App in November 2016 (Table). The app aims to foster children’s skills and provide them with new information that heightens their awareness on the importance of adopting a healthy lifestyle to avoid childhood obesity. This unique, culturally relevant app has received thousands of YouTube views, and require careful monitoring and evaluation to ascertain its impact in raising awareness and influencing attitudes and practices related to childhood obesity. However, more emphasis on the role of obesogenic environments^54^ in increasing the risk of childhood obesity is required in UAE and in neighboring nations.

## Conclusion

The prevalence of obesity among UAE adults is at least double those of children. The most effective way of sustainably addressing obesity in UAE is to start with reducing the prevalence in children, who have up to 80% risk of becoming obese in adulthood. This study sought to analyse the frequency of UAE’s leading newspapers’ coverage of childhood obesity and examine how obesity is framed in terms of sources of information, defining the problem, attributing causes and presenting solutions. UAE newspapers are currently playing a constructive role in contributing to efforts to address childhood obesity through raising awareness and reporting on efforts by stakeholders to address this health issue. Newspaper journalists can contribute even more to addressing childhood obesity through more in-depth comparative analysis of government’s childhood obesity policies and programs. Social media platforms such as Facebook, Twitter, and YouTube have the potential to aid UAE’s health authorities in disseminating childhood obesity information and implementing related initiatives nationally. More evidence is needed to develop best practices for adapting social media platforms for controlling childhood obesity.

## Ethical issues

Not applicable.

## Competing interests

Authors declare that they have no competing interests.

## Authors’ contributions

NA developed the research concept and scope. SA and AA reviewed internet and newspaper sites to access all data used in this study. All authors contributed equally to data analysis and writing up the manuscript.
